# Ethical conflicts during the process of deciding about ICU admission: an empirically driven ethical analysis

**DOI:** 10.1136/medethics-2020-106672

**Published:** 2021-01-05

**Authors:** Mia Svantesson, Frances Griffiths, Catherine White, Chris Bassford, AnneMarie Slowther

**Affiliations:** 1 University Health Care Research Center, Faculty of Medicine and Health, Örebro University, Örebro, Sweden; 2 Warwick Medical School, University of Warwick Warwick Medical School, Coventry, UK; 3 Patient and Public Representative, Trustee, ICUsteps - the Intensive Care Patient Support Charity, Coventry, UK; 4 Department of Anaesthesia, Critical Care and Pain, University Hospitals Coventry and Warwickshire NHS Trust, Coventry, Coventry, UK

**Keywords:** applied and professional ethics, clinical ethics, decision-making, moral Status, resource allocation

## Abstract

**Background:**

Besides balancing burdens and benefits of intensive care, ethical conflicts in the process of decision-making should also be recognised. This calls for an ethical analysis relevant to clinicians. The aim was to analyse ethically difficult situations in the process of deciding whether a patient is admitted to intensive care unit (ICU).

**Methods:**

Analysis using the ‘Dilemma method’ and ‘wide reflective equilibrium’, on ethnographic data of 45 patient cases and 96 stakeholder interviews in six UK hospitals.

**Ethical analysis:**

Four moral questions and associated value conflicts were identified. (1) Who should have the right to decide whether a patient needs to be reviewed? Conflicting perspectives on safety/security. (2) Does the benefit to the patient of getting the decision right justify the cost to the patient of a delay in making the decision? Preventing longer-term suffering and understanding patient’s values conflicted with preventing short-term suffering and provision of security. (3) To what extent should the intensivist gain others’ input? Professional independence versus a holistic approach to decision-making. (4) Should the intensivist have an ongoing duty of care to patients not admitted to ICU? Short-term versus longer-term duty to protect patient safety. Safety and security (experienced in a holistic sense of physical and emotional security for patients) were key values at stake in the ethical conflicts identified. The life-threatening nature of the situation meant that the principle of autonomy was overshadowed by the duty to protect patients from harm. The need to fairly balance obligations to the referred patient and to other patients was also recognised.

**Conclusion:**

Proactive decision-making including advance care planning and escalation of treatment decisions may support the inclusion of patient autonomy. However, our analysis invites binary choices, which may not sufficiently reflect reality. This calls for a complementary relational ethics analysis.

## Introduction

There has been a call in the medical ethics literature for methods of bringing philosophical and social research traditions together to explore the ethical dimension of the clinical context and to derive normative claims from empirical situations.^1 2^ In this paper, we describe the application of an ethical analysis approach used in the clinic^3^ on empirical data from a UK project of clinical decision-making around referral and admission to intensive care.^4^ We also draw wider normative recommendations to support decision-making in this situation.^1 5^

We define ethically difficult situations as ‘*situations in which you experience unease or uncertainty of what is right or good to do or are in disagreement about what should be done*’. 6 In the literature, descriptions of these situations in relation to intensive care unit (ICU) are usually around headline life and death issues regarding patients already admitted^7^ and there is little literature on decisions to refer or admit to ICU.^8 9^ To address this gap, a multidimensional mixed methods project ‘Understanding and improving the decision-making process surrounding admission to the intensive care unit’ was conducted.^10^ The initial ethnographic study of decision-making around admission to ICU revealed a decision-making process that included the following steps making and receiving a referral, gathering information about and simultaneously caring for the patient, making the decision and simultaneously caring for the family, implementing the decision and dealing with consequences.^4^ In addition to explicit ethical dilemmas around balancing burdens and benefits of treatment, we identified more or less implicit ethically difficult situations at each step of the timeline, which called for ethical analysis.

The ‘Dilemma method’ was initially described and developed as a conversation method to use in moral case deliberation, a model of clinical ethics support in the Dutch healthcare system.^3 11^ Its theoretical underpinning is in hermeneutics.^12 13^ The core element is the focus on *one* concrete dilemma and the experiences of participants in daily practice. Rather than using an a priori ethical framework, ‘*knowledge and moral judgments are constructed and examined in and with practice itself*’.^3^ We have adapted this model as a research method for empirical ethical analysis. We use the method to focus on an individual ethical dilemma and then extend our analysis to make more general normative claims about similar types of situation drawing on ‘wide reflective equilibrium’.^1 5 14^

In summary, this paper makes two novel contributions: (1) consideration of implicit ethical conflicts during the decision-making process for admission to ICU and (2) the use of a clinical ethics support method in the analysis of research data. Thus, the aim of this paper was to analyse ethically difficult situations arising during the process of deciding whether a patient should be admitted to ICU, using empirically driven ethical analysis.

## Method of the ethnographic study

### Design

This is a focused ethnographic study.^4 10^

### Setting and sample

Six hospitals in England were sampled for diversity of type of hospital and ICU unit size. A researcher (MS) shadowed intensivists receiving acute referrals for ICU admission from medical, surgical and emergency departments, observing decision-making processes regarding 45 patients. Ninety-six post observation interviews were held in total with intensivists (ICU consultants and ICU registrars), referring doctors, outreach nurses, patients and family members (table 1).

**Table 1:** Demographic data of participants in the observed decision events and dropout reasons

Patients			Family		
	Observed	Interviewed		Observed	Interviewed
N	45	3	N	42	13
Female/male, N	27/19	1/2	Female/male, N	25/17	6/7
Age mean (range)	61 (19–94)	–	Relation to patient		
			Daughter/son	23	6
Reasons for not being interviewed	15		Parent	6	2
Death	2		Spouse/partner	16	4
Declined participation in hospital/declined later or lost contact	4/8		Daughter-in-law	3	1
Missed to be approached by MS or hospital research nurse	13		Sibling/niece/grandchild	7	–
			Reasons for not being interviewed		
			Declined	6	
			Not asked due to distress	7	
			Not available/no response to initial information	9	
			Other family interviewed instead	7	

Clinicians					
	Total N=80	Consultant	Registrar	Junior doctor	Outreach nurse*
Female/male, N	25/55	6/26	10/25	3/3	6/1
Age mean (range)	42 (25–60)	46 (34–60)	34 (27–52)	31 (25–40)	44 (28–60)
Years experience in current specialty, mean (range)	8 (0.1–22)	10.0 (0.5–22)	4 (0.1–20)	2 (0.2–4)	8 (0.3–15)
Years experience since graduation, mean (range)	17 (1–41)	23 (10–36)	10 (4–29)	4 (1–12)	44 (28–60)
Specialty					
Intensive care	55	23	19	0	7
Medical specialties	15	7	8	6	0
Surgical specialties	4	1	3	0	0
Acute/emergency medicine	6	1	5	0	0

*Critical care outreach nurses, supporting ward nurses and doctors who are caring for acutely ill inpatients.

### Data collection

Observation sessions took place within a 3-week period at each hospital. During an observation period, the researcher (MS) accompanied intensivists responding to referrals they received. The intensivist introduced the researcher to the patient and/or their family member and sought their permission for the researcher to observe the conversation. Field notes were taken during the observations. Semistructured interviews with clinicians and family members were carried out by MS as soon as possible after the observed decision, and with patients in their homes 3 months after decision. Clinicians were asked to give their account of the observed decision-making process and also their experience of the decision-making process more generally. Patients and family were asked to describe their experience of the situation. All participants provided written consent for participation in the interviews. The digitally recorded interviews varied in length between 5 and 80 min (mean 17 min) and were transcribed verbatim.

### Qualitative analysis

Observation field notes and interview data were analysed using the Framework method,^15^ facilitated by the software program NVivo V.11 (QSR International). A detailed description of the analysis is provided elsewhere.^4^ Codes relating to ethically difficult situations were extracted from the main analysis and selected for ethical analysis as described below.

## The ethical analysis

To provide an ethical analysis of our identified ethically difficult situations, we adapted the ‘Dilemma method’ used in moral case deliberation^3^ (see table 2). The process of analysis includes presenting the situation (ethnographic storytelling^10^), formulating the moral question, formulating options, describing perspectives and values at stake, identifying the ethical conflict(s), balancing between ethical principles and modifying options (table 2). For the balancing, we drew on ‘wide reflective equilibrium’^5 14^ in order to make general claims (table 2).

**Table 2:** The ethical analysis process inspired by the ‘Dilemma method’^3^ and ‘wide reflective equilibrium’^5 14^

Analysis process	Adapted Dilemma method for research purposes	Corresponding steps in the Dilemma method^3^
Presenting the situation	Prior to the deliberation, MS presented written descriptions of four recurring ethically difficult situations from an observer perspective (Boxes 1–4). The situations were derived from between 1 and 4 of the 45 cases observed in the ethnographic study, and presented in a storytelling format.^10^ The condensed descriptions originated from field notes and interviews.	**Step 2. Presentation of the case.** The case presenter is asked to provide short but thick description of the facts about a situation where he/she has experienced a moral issue at stake. The focus is on the ‘moment of heat’, which implies a situation experienced strongly as a moral dilemma within a specified timeline.
Formulating the moral question	Formulation of the moral question arising from the situation and two opposing normative options emerged through dialogue in the analysis group where the perspectives of the case presenter/observer and the interviewees were considered. MS also tried to bring the perspective of the patient. MS presented a preliminary analysis including field notes and interview transcripts (figure 1 and online supplemental file) which was scrutinised, shifting between formulation of the moral question/options and clarifying questions, facilitated by AS.	**Step 3. Formulating the moral question and Step 4. Clarification.** The case presenter sketches the case, making the moral question explicit, for understanding of what is morally at stake. The facilitator helps to formulate the question by asking questions and also helps to formulate the dilemma as two opposing options (A and B). The facilitator invites participants to ask questions for clarification. The aim is to reconstruct the situation and to foster an understanding by imagining themselves in the shoes of the case presenter at the moment of heat.
Identifying perspectives, values and at stake	We identified values related to the perspectives of those involved in the situation (figure 1). This was an iterative process shifting between articulated arguments and norms identified in the research data that reflected values that supported each specified normative option (A or B). Quotations from interviews and field notes were used to tease out the perspectives and values and demonstrate trustworthiness of the analysis (online supplemental file).	**Step 5:** Analysing perspectives, values and norms. The facilitator asks the participants to jointly construct a list of relevant values and norms for each perspective (stakeholder). This includes values and norms either supporting option A or B.
Identifying the ethical conflict	The group identified which values were under pressure, including tensions both within and between values, and developed a joint formulation of the ethical conflict/s.	**Step 8. Dialogical inquiry.** The facilitator helps the participants to find the values under pressure, whether both options are based on the same values or different values for the same option. Here the participants learn to see others’ perspectives.
Balancing between options and ethical principles	In finding justifications for option A and B, we added ‘wide reflective equilibrium’^5 14^ in order to make general claims. We modified our considered judgements through an iterative process seeking coherence from a ‘wide’ scope of moral views (our own and those of participants), principles, theories and facts, moving the options closer to each other=equilibrium.	**and Step 7. Individual choice.** The facilitator asks each of the participants to give personal viewpoints, answering questions about justifications for choosing option A or B. This enables learning from each other’s reasoning.

10.1136/medethics-2020-106672.supp1 Supplementary data

Box 1 **The ethically difficult situation: observed and experienced social conflict during a referral between the intensivist and the referring doctor, drawing on composite cases**

The intensivist on call receives a phone call in the hospital corridor on his way from reviewing a patient to his consultant to ask for advice. It is a junior doctor on a medical ward, speaking rapidly, saying that her patient is breathless and that he needs to be admitted to ICU. The intensivist asks about the patient’s blood gas results, but there are none. The intensivist experiences the referral as unclear and impolite and is not keen to review “*I wasn’t certain why that referral was made … She should give a clear reason for calling and she should be able to explain what the concerns are … demanding a service rather than asking for an opinion”*. The referring junior doctor experiences the intensivist as dismissive and resistant to reviewing the patient: “*I said this patient is unstable, and I think it could have got maybe a little slicker if the intensivist had been more receptive”*. She feels stressed, having to manage the ward on her own without senior support and she is in need of competent support. The doctors seem to have different perceptions about what should be expected of each other.

Extracts from the decisions number 5 hospital 1, number 6 hospital 3 and number 2 and 3 hospital 5

Box 2 **Ethically difficult situation 2: observation of intensivist gathering information**

The intensivist is called to the resuscitation room in the emergency department. The patient is half sitting with bare chest on the stretcher and repeatedly tries to rip off the oxygen mask. She tries to lean forward, she is sweating, out of breath and her eyes seem to express despair. The environment is chaotic, the resuscitation room is full of patients, and many staff are performing medical procedures; you can hear a patient screaming on the other side of the curtain. The intensivist approaches the patient and asks questions about her previous treatment and mobility in a loud voice; such as ‘*Do you get up and down the stairs? Can you walk on the flat without being out of breath?*’ The patient answers yes to all the questions (later the intensivist learned from the family that the answers were incorrect). Then the intensivist decides that an arterial gas is needed and begins the procedure to insert an arterial catheter, but it is problematic. The patient groans and wriggles: ‘*Please let me go’*. The intensivist turns to me: *‘I really need a gas’*. Staff are holding the patient and repeat: ‘*Keep still, please, please, don’t move’*. An emergency doctor shouts: “*Give her morphine*”, but the intensivist responds that he wants to prevent intubation. After a while, the intensivist approaches the patient at the headboard again. He asks: ‘*Have you thought about what happens if you get worse?*’ No answer. ‘*Do you want dialysis?’* The patient nods. ‘*Would you be put to sleep on a breathing machine*?’ Patient rotates her hands, eyes widening. The intensivist calls his consultant and they discuss the need for a scan before a decision is made, but also that ICU may be the best comfort for the patient at this stage. Then, her granddaughter is guided in by a nurse, the patient smiles, calms down, nods off, the pulse on the screen lowers.

Extracts from the cases number 4 hospital 2, number 4 hospital 3, number 1 hospital 4 and number 5 hospital 5

Box 3 **Situations 3.1 and 3.2: observation and experiences of different kind of input before decision-making**

3.1 On a medical ward, the intensivist is standing at the bedside looking at the patient, while an anesthetic registrar supports the patient’s breathing with a bag mask. The patient is wheezing; the atmosphere is busy with lots of staff around the bed. The intensivist says they will admit him and later comments to me ‘*he’s a critical care decision’*. He calls ICU to inform them which patient to transfer as ICU is full. Meanwhile, the medical consultant that referred the patient conducts the ward round and I learn later from her that the patient has several co-morbidities and a low functional status. Observer: “Did you communicate with the doctor on the ward?” Intensivist: “No”. Observer: “Not afterwards either?” Int: “No”. He says it depended partly on the time constraint, partly that the expertise needed for treatment in this case was that of critical care and lastly, consultants in this specialty don’t usually communicate with them regarding patients admitted to ICU. The intensivist adds that there is a danger in asking the family as they may “*have very strong feelings … their hopes are unrealistic; they want everything done”*. Decision 3, Hospital 2

3.2 In the resuscitation room, a patient is lying flat on the stretcher with his niece sitting by his side. He is looking pale, staring ahead and now and then groaning, as if in pain. The intensivist tries to make contact with him, but he does not seem present. The intensivist asks the patient’s niece what has happened. She mentions results from a scan and asks whether he knows about it. The intensivist: “*Sorry, I haven’t read the scan report*”. It looks like the niece knows the results and I get the impression it is a bad result. After talking to his consultant, he calls the surgical junior doctor who referred the patient: “*I have seen the patient, talked with his niece and talked to my boss (ICU-consultant) and unfortunately we need a scan first to see*”. I learn later that the surgical registrar has called her consultant at home and in the coffee room on ICU, I listen to a telephone conversation on loudspeaker between the consultants. The surgical consultant contributes with expertise about the disease. They decide to meet later in the resuscitation room to assess the patient together. Afterwards they talk with the niece in a separate room. She tells them that she remembers that the patient said when his wife was going through intensive care: *‘Don’t ever let me go through this’*. The referring junior doctor concludes ‘*It’s gold dust when people can actually tell you what the patient would want’*. Decision 1 Hospital 1, number 8 Hospital 2 and number 4 Hospital 5

Box 4 **Situation 4(c) describes a patient assessed as too ill for ICU and likely to die. Situation 4(d) describes a patient not requiring ICU just yet, but if getting worse being too ill to benefit from ICU**

4(c) The senior intensivist goes down to the ward where a decision not to admit has been made, conveyed earlier by the junior intensivist. (Senior intensivist): “*I was fairly clear that the patient would not benefit from coming to have any intensive care therapy but the patient still had needs and the patient in this case had end of life care needs … The referring junior doctor was there, so we had a joint discussion together, we talked about whether other palliative treatment for his tumor symptoms might be of any benefit. This was getting out of hours now, I think it was about 6pm. … we agreed that we could probably manage a sort of sedation which is possible to administer on a ward. … I also tried to sort of emphasise I thought we should be thinking more about end of life issues, this gentleman’s life is going to be measured in months, and so starting to think about where he’d want to die, getting his views about his own end of life care, obviously that wasn’t possible at the time because he was fast asleep. The moment this gentleman came into hospital they probably knew he had a terminal tumor and yet we wait until there’s a crisis before thinking about what the future truly holds”*. The senior intensivist then has a long conversation with the family, before he goes home. Decision 3 Hospital 5.

4(d) It is evening on an orthopaedic ward and the intensivist has just made the decision to not admit the patient. The intensivist thinks that the patient currently does not need intensive care treatments, yet severely ill. He says: ‘*My main issue is that she has been in hospital a longtime and she is not thriving … not fit for surgery, dialysed for a long time, look at the co-morbidities, we have done enough for her*”. Still he feels that the patient’s current condition needs to be addressed. There is no doctor on the ward, but the referring doctor had previously expressed that he will be *“entirely guided by the (intensivists) decision”*. The intensivist catches a nurse on the go and informs them that the patient needs prescriptions regarding blood pressure medication, fluids and monitoring. The nurse looks scared and wonders whether he (the intensivist) can prescribe and speak to the patient’s family. The intensivist: “*Please phone your doctor on call”*. Decision 6 Hospital 6

**Figure 1:**
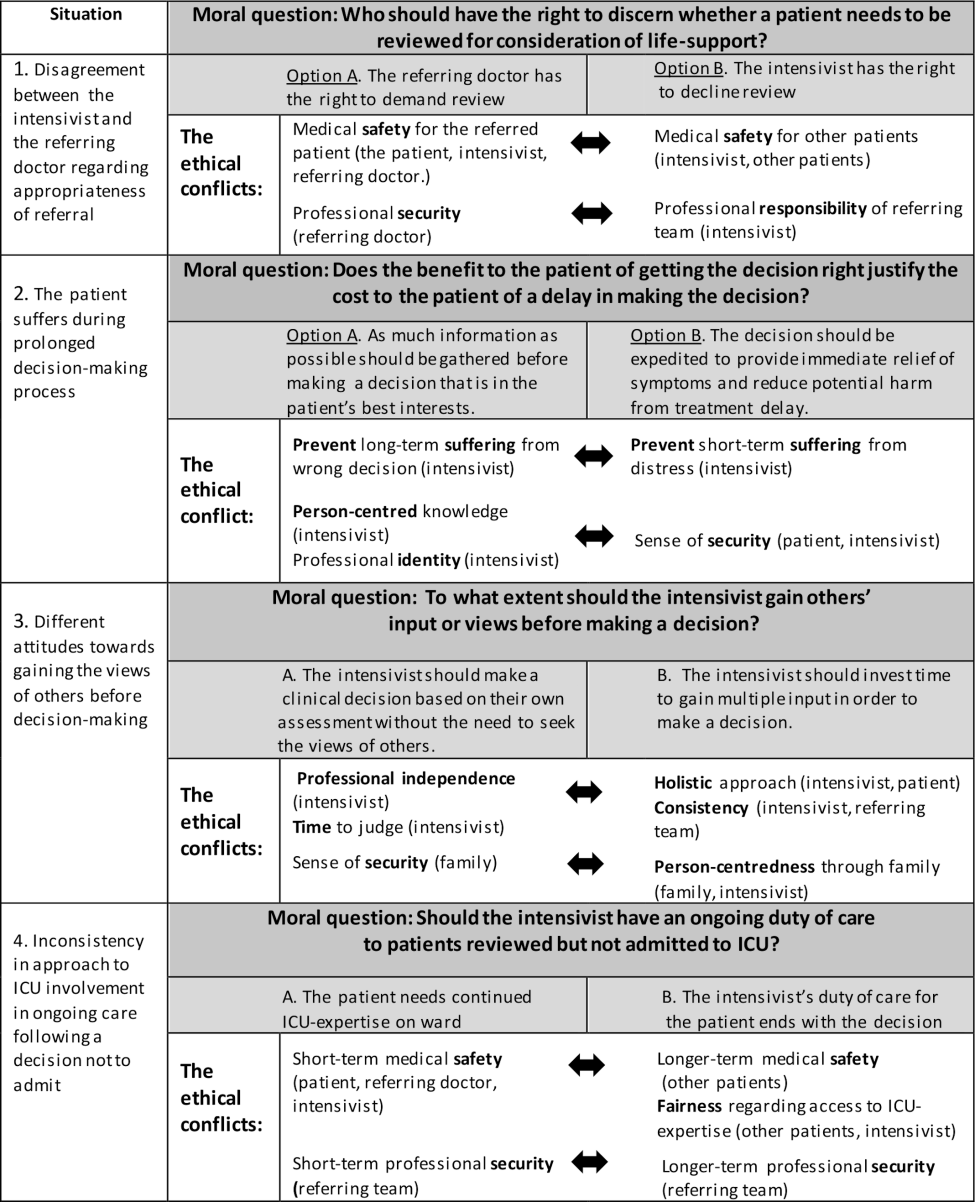
Summary of the key ethical conflicts in the ethical analysis; values conflicting *within* and *between* different perspectives (stakeholders) during the decision-making process. ICU, intensive care unit.

AS (ethicist with background in general practice) facilitated the iterative moral case deliberation (MCD) meetings. The case presenter was MS (the ethnographic observer, ICU nurse, ethicist), who brought in a total of four ethically difficult situations, captured along the timeline of the decision-making process. The other participants were an intensivist (CB), a former ICU patient (CW) and a general practitioner/social scientist (FG). Stakeholders in the analysis were mostly clinicians, as few patients and family member interviews were obtained, but MS tried to represent their perspective, based on observation.^4^

### Situation 1: disagreement between the intensivist and the referring doctor regarding the appropriateness of a referral

#### Presenting the situation

This situation was embedded in observed social conflicts between intensivists and referring doctors (box 1).

#### Formulating the moral question

The initial formulation of the moral question, “Should all referrals be respected?” developed eventually through deliberation into the following question and opposing options: Who should have the right to decide whether a patient needs to be reviewed for consideration of life-support? Two alternative options:

- The referring doctor has the right to demand a review.
- The intensivist has the right to decline review (figure 1).

#### Identifying perspectives and values at stake

For this situation, the perspectives of referring doctors and intensivists were drawn directly from the data but the perspectives of patients were inferred and were advocated for by the clinicians. The referring doctors mainly supported option A but the intensivists’ perspectives were more mixed, representing views supporting both options. The values and associated arguments supporting each perspective are presented below, but see also further quotes and field notes in online supplemental file, table 3.

*Medical safety* implied protection from physical harm, either associated with treatment or from having an unrecognised deterioration. From the perspective of the referring doctors, medical safety of their patient was seen as reflecting the implicit value that their patients would hold in wishing to be kept safe. Safe could either mean an intensivist’s watchful eyes or to be moved to ICU. They argued that they were in the best position to assess this as they had seen the patient and had the relevant information to be able to make a judgement about the need for referral. They were concerned that an intensivist who had not seen the patient would make assumptions and therefore not adequately consider the potential benefits and harms of admission “*he picked up on why I was calling them… ‘Exactly how old is the patient?’ Oh it frustrated me. … I think you need to see a patient before you can write them off”* (online supplemental table 3).

For those intensivists whose perspective appeared to support option B, the value of patient safety was perceived more in terms of representing other patients, as the gatekeeper to access for scarce ICU resources in relation to others who may be in greater need of their attention. Many intensivists argued for more informative referrals, in order to prioritise which patients needed their attention most urgently: “*it was quite annoying because if she (referring doctor) had assessed the patient and done the gas, it wouldn’t have occurred … we pulled the ITU doctor away from doing something else”* (online supplemental table 3). However, others suggested that insisting on a well-prepared referral (rejecting unclear referrals) might put critically ill patients at risk, and preferred to rely on the referring doctor’s judgement.

*Professional security* (figure 1 and online supplemental table 3). Referring doctors valued the experienced security of having the intensivist coming to see the patient, and of being able to meet the intensivist face to face. This was also recognised by intensivists *“my reasons for being a soft touch is that I think that by the time the wards have called us they’ve tried many things*” (online supplemental table 3).

*Professional responsibility.* Intensivists emphasised the need for referring doctors to assume professional responsibility, expecting them to assess and treat patients under their care competently and not offload this responsibility to the intensivist by premature or unnecessary referrals. They also needed referring doctors to provide accurate and comprehensive information to enable the intensivist to prioritise which patients most needed their attention. Intensivists felt they had the right to decline a review that lacked the structure of the communication tool SBAR (Situation, Background, Assessment, Recommendation). They also expressed irritation at what was perceived as manipulation of the presentation to secure an ICU review by describing patients as more ill than they actually were: *“they always say that they are tiring”* (online supplemental table 3).

#### Identifying the ethical conflicts

The MCD group identified an internal conflict within the value of medical safety, experienced by the intensivist group and regarding the referred patient as opposed to other patients. There was also an identified tension between the referring doctors’ need for professional security and the intensivists’ need for the referring team to assume responsibility (figure 1). The group identified that the key ethical principle at play was that of minimising or protecting patients from harm (non-maleficence). The duty to protect from harm was in tension because of the potentially competing claims or interests of the patient being referred and other patients requiring the attention of the intensivist. Concerns about professional security and responsibility were also driven by the concern to protect patients from harm.

#### Balancing between options A and B

The considered judgement of the MCD group was that the intensivist has an obligation to review all referrals in person (option A), but that certain criteria should be in place to support this obligation. Drawing on wider ethical principles, we considered that the key principle at stake in addition to non-maleficence is justice; that intensivists must balance the needs of different patients and that best use should be made of the joint resources of referring and ICU teams. This would imply that certain criteria for referrals are met (moving closer to option B). First, the referring doctor needs to make a reasoned and informed case for the need for review. The use of a referral pro forma such as the SBAR tool would assist in this process. Second, the intensivists can postpone a review if they are too busy dealing with patients who they judge to be sicker than the referred patient based on the information provided; and third, doctors should be persuadable by the reasoned argument of another doctor (ie, not intransigent). Our suggested resolution protects the safety of patients and the professional security of referring doctors while placing a responsibility on both referring doctors and intensivists to provide reasoned justifications for their actions, and enables intensivists to make considered judgements about how to allocate their expertise between patients.

### Situation 2: the patient suffers during a prolonged decision-making process

#### Presenting the situation

The observer (MS) recognised that there was a tacit understanding among clinicians that patients may suffer when the decision-making process is prolonged as the intensivist deliberates whether ICU treatment would be beneficial for the patient (box 2). MS observed suffering related to painful procedures, withholding of symptom relief and questions that seemed to distress the patient.

#### Formulating the moral question

Two initial moral questions were posed by MS: “Is it right to ask life and death questions to a patient struggling to survive?” and “Must the patient endure suffering between referral and decision? Following discussion, the moral question was articulated as: Does the benefit to the patient of getting the decision right justify the cost to the patient of a delay in making the decision?

- As much information as possible should be gathered before making a decision to ensure that it is in the patient’s best interests.
- The decision should be expedited to provide immediate relief of symptoms and reduce potential harm from treatment delay (figure 1).

#### Identifying perspectives, values and norms at stake

No one involved in these situations explicitly expressed concern about suffering to the patient caused by gathering information and delayed decisions, but we see the patient and the intensivist as the key stakeholders in this situation (figure 1 and online supplemental file, table 4).

For *the patient*, a sense of physical and emotional security appeared to be the key value from their perspective. In these situations, most patients seemed to lack capacity to hold a meaningful conversation about their treatment. Patients expressed afterwards that they preferred to let the doctors make the decisions *“the doctors know what they’re doing. It’s no good saying to me, ‘What do you think about it?’ because I haven’t got a clue*” (online supplemental table 4). Being able to trust their doctor was important and contributed to a sense of security. MS observed that patients were less distressed when the doctor was calm, experienced and competent, and by the presence of a familiar person.

*Intensivists* expressed a concern to prevent long-term suffering by reducing the risk of an incorrect decision being made to admit the patient to ICU or provide invasive treatment such as ventilation when this may not be in their best interests. This meant avoiding sedation to keep the patient alert to gain information: “*she was agitated, thrashing around, but we weren’t keen to intubate her*”. There were however examples of intensivists admitting patients before they had gathered all relevant information in order to *prevent* short-term suffering. They recognised that there was a risk of suffering in delaying a decision until information was available, and prioritised expediting immediate relief of symptoms and postponing painful procedures (online supplemental table 4). Other intensivists delayed decision by trying to gather person-centred knowledge from the patient. This meant trying to understand the patient’s values, mostly questions about health-related quality-of-life such as functional status, but also patient’s wishes. A few clinicians recognised or cited a legal duty to involve patients in the discussion of ceilings of care at the first encounter.

Additionally, the value of professional identity seemed important as a reason for prioritising collection of information, from the patient and from clinical investigations, particularly for the junior intensivists. It was important to them to make sure that expected investigations had been done and that the decision was made on best available evidence. This was expressed as a sense of professional competency, but also self-protection against being judged by peers: “*if you admit them to ICU and they fail to benefit, you can feel as if you’re being judged by your peers as, as to have made a wrong admitting decision*.”

#### Identifying the ethical conflicts

Implicit value conflicts experienced by the intensivists were those between preventing long-term suffering, respecting the patient’s values and protecting their professional identity, and preventing short-term suffering/providing security for the patient (figure 1). The MCD group identified the key ethical conflict in terms of how to do the most good for the patient. Will more good be achieved if the benefit of initiating invasive treatment is accurately predicted and the patient’s views are understood before the decision or will more good be achieved by immediate treatment to relieve short-term harm and support a sense of security?

#### Balancing between options A and B

Evaluating the level of beneficence between the approach in option A and option B will depend on the individual case, but overall the group considered that the ethical priority of maximising benefits favours option B. The principle of respect for autonomy in this situation is bound up in the balancing of beneficence where respecting patient’s wishes contributes to their overall benefit. Acknowledging that patients may lack the ability to exercise their autonomy in these situations (acknowledged also by the group’s patient member), the focus should be on the patient’s lower level needs of physiological and ‘*safety/security needs*’.^16^ However, once again the group set limits on the selected option. In responding to immediate physical and emotional needs, it is important to recognise the potential harms of these actions, and to mitigate these by early review when the situation is more stable, further information is available and/or the patient is in a position to contribute to decision-making. This approach is an established management option; often referred to as a ‘trial of intensive care’ (conditional life-support and symptom relief).

### Situation 3: different attitudes towards gaining the views of others before decision-making

#### Presenting the situation

Usually, the ICU consultants acted as the formal decision-makers, but there was variability regarding their attitude to involving others to provide information or to express their views. The observer (MS) perceived most patients were too sick to speak to the intensivist, so the other stakeholders presented are the referring team and box 3 family.

#### Formulating the moral question

This situation generated the most deliberation in terms of formulating the moral question of all the situations identified for analysis. Views differed over whether the family had a role in making the decision or their role was limited to providing information. Our interview data with families suggested that this was unclear to them. MS posed two preliminary questions: “Is being a sole decision-maker without seeking the views of others right?” and “Is the variability in intensivists’ attitudes towards involving others in decision-making acceptable?” The final formulation of the question was: To what extent should the intensivist gain others’ input or views before making a decision?

- The intensivist should make a clinical decision based on their own assessment without the need to seek the views of others.
- The intensivist should invest time to gain multiple perspectives in order to make a decision (figure 1).

#### Identifying perspectives and values at stake

Intensivists and families were split in their views on this situation while referring doctors emphasised the importance of multiple perspectives (online supplemental file, table 5).

Some *intensivists* valued professional independence and emphasised their particular knowledge in understanding what critical care can and cannot be achieved. They thought other doctors and family members had unrealistic expectations, “*it’s not a magical land where everything will be fixed miraculously; there are limits to what can be done*” (online supplemental table 5). They emphasised the norm of making a decision that is in the best interests of the patient and considered that they are best placed to do this. They also considered themselves to be best placed in terms of time and that referring doctors may not be familiar with the patient and also be delayed if providing care to other patients *“I have the luxury of time when I go and see a patient … 40 min, when considering a non-admission*.”

Other intensivists sought to gain a holistic view of the patient by including other views. They appeared to value consistency of decision-making, involving others to protect against cognitive bias and to mitigate against the problem of different intensivists having different thresholds for admission. Intensivists described themselves as being a ‘*hawk*’ or a ‘*dove*’ (online supplemental table 5).

*Referring doctors* conveyed a need to share their perspectives with the intensivist about the referred patient to gain a more rounded view of the situation and what was possible in terms of treatment: “*a second pair of eyes and ears is very helpful and the interaction today has been gleamed*” (online supplemental table 5).

Fo*r family* members, being able to trust the doctor to make the decision gave them a sense of security, as they felt being in an emotionally unstable state, as expressed by a patient’s son:

a bit of turmoil and… you don’t take in everything they’re saying and it would be better if they said, ‘Right this happened, we are now moving her’.

Additionally, family members may not know the patient’s wishes or feel comfortable talking about it. One patient interviewed described trying to talk about end-of-life with her son: “*he says don’t talk so silly! He doesn’t like talking about things like that*” (online supplemental table 5). In these circumstances, avoiding discussion with the family could be seen as protecting them from added distress. However, some family members advocated for family input to convey their views to the clinicians, enabling the patient’s voice to be represented: “*… if dad had strong views and I knew about it, then I do think it is something that should be asked*” (online supplemental table 5).

#### Identifying the ethical conflicts

We identified a conflict between two conceptions of the intensivist; independent professional expert or one perspective contributing to a holistic view of the patient, where other stakeholders contribute with their perspectives. This conflict arises from different views of where expertise and agency lies. These could be described as: The ‘critical illness exceptionalism’ model that assumes expertise resides in intensive care for critically patients, versus the ‘continuum of disease’ model that critical illness resides along a spectrum of disease that is within the domain of the referring doctor. There is a parallel conflict between the medical model of best interests, where the clinical expert is best placed to make the assessment, and the holistic, person-centred model, where best interests can only be determined by taking account of the patient’s perspective.

In addition, intensivists experience an ethical conflict between protecting the family from harm by not adding to their distress, and respect for patient autonomy by identifying the patient’s values through conversation with the family. Between the intensivist and the referring doctor, the ethical conflict arises from different models of where expertise and agency lies.

#### Balancing between options A and B

The group considered the wider principle of beneficence. The clinical expertise and experience of the intensivists is clearly crucial regarding what ICU can achieve, but this needs to be informed by wider considerations to truly benefit the patient. This can only be achieved by including information and views from other sources in particular information on the patient’s wishes and values but also the experience of the referring team on the patient’s illness trajectory and prognosis. Thus, favouring option B implied merging the models of ‘critical illness exceptionalism’ and ‘the continuum of disease’. This option would also reflect the principle of justice, supporting the values of consistency of decision-making process. However, the duty to protect a person from harm would also set limits to a holistic approach, for example, in an emergency situation where there is no time to obtain other views and the immediate decision is based on acute clinical need.

### Situation 4: inconsistency in ICU involvement in ongoing care following a decision not to admit

#### Presenting the situation

In some hospitals, it seemed to be routine to follow up patients assessed as not requiring immediate admission to ICU, while in other hospitals, it depended on the individual intensivist’s approach. Four groups of patients were identified in this category of not requiring immediate admission to ICU, namely, patients who were (1) too well for ICU and unlikely to need it in the immediate future; (2) currently too well for ICU, but will possibly need it soon; (3) too ill for ICU and likely to die even with ICU treatment; and (4) not requiring ICU just yet, but would not be a candidate for ICU should they deteriorate because of their underlying condition(s). The last two situations seemed more likely to result in ethically difficult situations in terms of ongoing review (box 4 ­).

##### Formulating the moral question

The MCD group achieved agreement on the moral question in this situation more quickly than in the other situations: Should the intensivist have an ongoing duty of care to patients reviewed but not admitted to ICU?

- The intensivist’s duty of care extends beyond the decision not to admit.
- The intensivist’s duty of care for the patient ends with the decision (figure 1).

##### Identifying perspectives and values at stake

Here, there were different perspectives both among intensivists and referring clinicians (online supplemental table 6).

Some *intensivists* felt strongly that they had a responsibility for severely ill patients even if they were not admitted to ICU: *“we don’t restrict intensive care even if we don’t have room in our ICU, I would arrange for that patient to receive appropriate care wherever”*. They also described the difficulty in anticipating the illness trajectory for patients initially judged as too well for ICU. The key value expressed in this perspective was short-term medical safety, reflecting the duty to protect the patient from harm, ensuring deterioration was acted on and that relevant treatment was provided. Other intensivists expressed frustration over the referring team’s failure to acknowledge their ongoing responsibility for the patient. They felt that by continuing to advise on patient management, they were facilitating the weakening of competencies outside intensive care and putting future patients at risk. They seemed to value long-term safety for all patients, implying sufficient expertise and staffing to be able to care for severely ill patients.

*The referring team* expressed reassurance at having ongoing input from the intensivists for the referred patient, providing a sense of short-term professional security, particular for junior doctors who often felt unsupported. “*She was mirroring my own thoughts… had a pragmatic view of the future and… then communicating to me what her concerns were*.” The value seemed to have its roots in a concern for the medical safety of their patient, but also for preventing suffering, as intensivists also gave end-of-life support management (online supplemental table 6).

##### Identifying the ethical conflicts

We identified an ethical conflict between short-term benefit for patients who continued to be reviewed or have an ongoing treatment plan specified by an experienced intensivist, and the potential longer term harm for severely ill patients if demarcation of responsibility is not clear and the need for increased medical expertise on the ward is not recognised.

##### Balancing between options A and B

Our considered judgement was that the intensivist has a moral duty to ensure a plan is in place to provide safe care for the patient who is not admitted to ICU (option A). This is grounded in the wider principle of non-maleficence, which implies that valuing safety and security is supporting the moral norm of protection of harm both for patients and the referring team. Additionally, having accepted responsibility for this patient in so far as having met the other person’s suffering, reviewing their situation and considering what treatment is needed, a moral responsibility towards this person is generated. This moral claim of protection is grounded in the philosophy of Levinas, manifested in the face-to-face encounter in an asymmetrical relation.^17^ However, Levinas did not discuss moral responsibility towards other patients the intensivists have not met. Thus, this theory and the principle of non-maleficence needs to be balanced with the principle of justice, setting a limit to the duty for the reviewed patient, moving closer to option B. When there are time constraints, the duty should be articulated as setting out an initial plan and communicating this to the referring team, but then leave the responsibility with the referring team and move on.

## Conclusion

Safety and security were the key values at stake in the ethical conflicts during the ICU decision-making processes, with the overriding ethical principles of non-maleficence and beneficence. Given that intensivists have multiple patients; the value of fairness was at stake; how does a decision for this patient impact on obligations to other patients. The patient voice was seldom heard directly in our data, but when it was, supplemented by our observations, it suggested that people value safety but in a more holistic sense of feeling secure. In a life-threatening situation, clinicians and patients prioritise the need to protect, or be protected, from the harm of clinical deterioration over the principle of autonomy. This can be likened by a log metaphor. You are carried away by water and grasps at a log (the log represents others that best can interpret (family) and fulfil your immediate needs (clinicians)). When reaching calm water, you gain confidence to let go of the log and are able to swim to shore (capacity to take control of your situation and make autonomous choices). We suggest that security in this holistic physical and emotional sense should be recognised as an ethical value, with intrinsic property of worth in itself, not as a means to something else.

## Implications for the organisation

The conflicts we have identified have arisen through the different perspectives not being integrated in any design of the decision-making system, and healthcare professionals having different understandings of the roles and responsibilities of others and themselves. Much of this can be addressed through an organisation developing systematic and integrated pathways for this decision-making process. Our analysis also generated a further moral question at an organisational level: should the resource of intensivist expertise be increased or should the focus be on increasing expertise on wards?

We recommend preventive measures for avoiding ethical conflicts in all four situations. These include increased advance care planning, but also proactive decisions about ceilings of care. Advance care planning in primary care should be encouraged as this may prevent suffering during the waiting time for decisions in emergency departments and facilitate person-centred decisions. In hospital wards, there needs to be greater emphasis on ceilings of care decisions being made before a patient deteriorates. This would improve communication between referring teams and intensivists facilitating shared responsibility for decision-making between referring and ICU clinicians. There has been increasing emphasis on emergency care treatment plans in the UK in recent years,^18 19^ brought into sharp focus by the COVID-19 pandemic. Involving the patient’s family in discussion may also help to ensure safe but person-centred treatment. Furthermore, if a palliative care decision is made earlier, there is evidence of more beneficial effects on quality of life and symptom intensity compared with patients who are given standard care.^20^

Increased staff resources and expertise on referring wards may reduce the need for what some intensivists see as ‘inappropriate’ referrals. This could include critical care outreach services. Communication may be improved with the use of structured referral tools such as SBAR and consultant to consultant discussion of cases. Wider communication and shared learning, for example, discussion of shared cases at grand rounds, may enhance a holistic approach and promote cooperation between intensive care and other specialties.^21^

The patient’s sense of security in the critical situation could be enhanced by ensuring that a familiar person or a nurse is responsible for supporting them throughout the process, providing reassurance and ensuring that the patient’s perspective is not lost. The nurse may engender a sense of security, while at the same time preserve autonomy by posing the difficult questions to patients in a responsive way, acknowledging their vulnerability.

## Evaluation of the methods

This analysis has used a new approach to describe some of the ethical conflicts inherent when decisions are made about the care that critically ill patients should receive. It is also the first time to our knowledge that a moral deliberation method^3^ has been used as an analysis tool in research. A major strength is that the method links the research closely with clinical practice by using a method that is used in clinical practice rather than a more abstract theoretical framework. The Dilemma method adopted is a pedagogical approach for clinicians to identify and evaluate ethical conflicts. However, decisions in reality are not always as binary as suggested by this approach. Therefore, we used wide reflective equilibrium to acknowledge and accommodate the range of values identified, and thus shaped a modified recommended solution. However, it is possible that our analysis may not have sufficiently acknowledged the relational element of the decision-making process. In the qualitative analysis of the ethnographic study, we also identified both good and bad communication between clinicians and with patient/family. A complementary relational ethics analysis may yield further insights into this challenging and complex area.

The use of data from observation and interviews, as well as the diverse perspectives of the analysis group, facilitated the imagining of moral issues and values in new ways. This has similarities to ‘imaginative ethics’ as described by Hansson,^22^ and seen as a complement to principle-based ethics. The specificity of the context during moral case deliberation enables different moral voices to speak.^22^ This facilitates the capturing of values on a more concrete level, which are then abstracted to ethical principles when balancing options. Wide reflective equilibrium enables a move to generalisable resolutions that can speak to systems and organisational activity.^5 14^

Other ethical analysis methods connected to clinical practice have been described including the actor model,^23^ the CME (Centre of Medical Ethics) six6-step model^24^ and the Ethics Deliberative framework (ETHICS).^25^ They share the basic steps of identifying the ethical issue, relevant facts, interests and values, and conclude with a course of actions. A major strength in using the ‘Dilemma method’ was the focus on identifying the moral question. Formulation of the questions dominated our iterative joint deliberations but once agreed, formulations of values and the ethical conflicts became clearer.

## Data Availability

All data relevant to the study are included in the article or uploaded as supplementary information.
