# Supplementary material for: Ethical conflicts during the process of deciding about ICU admission: an empirically driven ethical analysis
Source: J Med Ethics. 2021 Jan 5;47(12):e87. doi: 10.1136/medethics-2020-106672 (PMC8639921; doi:10.1136/medethics-2020-106672)
Supplement: Supplementary data [file medethics-2020-106672supp001.pdf]

Supplementary file

Table 3. Sources to show the ethical analysis' trustworthiness regarding clashing values for the right of review. Values= CAPITALS, arguments and norms attached to values=underlined, sources i.e. quotations (in *italics)* underpinning views and field-notes as responses to the question:
Who should have the right to decide whether a patient needs to be reviewed for consideration of life-support?

**A. The referring doctor has the right to demand a review**	**B. The intensivist has the right to decline review**
MEDICAL SAFETY FOR THE PATIENT<br>Referring doctors have an obligation to protect the patient from harm, therefore they should have the ability to make judgments: "*… he picked up on why I was calling them; he wanted me to stress the patient was 89 and he asked me to specify a few times, 'Exactly how old is the patient?' Oh it frustrated me. Because this senior is not quite well. I think you need to see a patient before you can write them off because we weren't asking for ventilator support we were asking for input."* Referring doctor, Decision 1, Hospital 2'<br><br>Requesting a well prepared referral might imply a risk for the patient: "*You're quite often being told that I shouldn't be called by junior team members but it's very difficult to tell if someone calls you for help and they can't get in contact with their consultant, you can't 'No I'm not coming until a consultant contact you', because that's probably too late and actually, it's very easy to go and see a patient on the ward … because it's the patient who would be the one who suffers*" Intensivist, Decision 2, Hospital 5	MEDICAL SAFETY FOR OTHER PATIENTS<br>Referral should be informative in order to prioritise: "*Any referral can be a little bit random at best. We always look at their past history in the background by the referral team, so that we can filter out the genuinely sick ones and also so that we can decide whether we need to address immediately.*" Intensivist, Decision 6, Hospital 1<br><br>Outreach nurse to the ICU-clinician: 'they shouldn't have called you' and there is a discussion whether the referral was needed or not, that it is not right that a senior house officer makes the referral. Field notes "*It was quite annoying because if she had assessed the patient and done the gas on oxygen, it wouldn't have occurred. The urgency was not required and we pulled the ITU doctor away from ITU when she might have been doing something else.*" Outreach nurse, Decision 2, Hospital 5
PROFESSIONAL SECURITY FOR THE REFERRING TEAM<br>"*We call them and they are always happy to come and have a look to a patient. … they are very good communication and they are happy to advise, to manage … so it's very good, professionally*". Referring dr. Decision 3, Hospital 4<br>Intensivist should be receptive to the needs of the referring team: "*My reasons for being a soft touch is that I think that by the time the wards have called us they've tried many things and they call us because they've run out of ideas or they've run out of resources and that resource might be personnel, might be monitoring, might be interaction*." Intensivist Decision 6, Hospital 4<br><br>Supporting the referring team needs will benefit the patient: "*I'd much rather know about these patients and go and see them myself and make a decision than just say, 'this isn't my problem'. … There is a patient at the end of it who has needs and unfortunately this gentleman had an incurable brain tumour*" Intensivist, Decision 3, Hospital 5	PROFESSIONAL RESPONSIBILITY OF REFERRING DOCTORS<br>Intensivist should not be a substitute for others' lack of time: "*From medicine there's an unwillingness to talk to the senior members of the team and ask for help before phoning intensive care. So it's usually goes side-ways before it goes upwards in the hierarchy*". ICU-consultant Decision 4, Hospital 1<br>*"The ED-registrar rang the medical registrar on call, they said, 'We're rushed off our feet can you ring the intensive care doctor on call?'"* Intensivist, Decision 6, Hospital 2<br><br>Referring doctors shirk from responsibility of informing: "I *treated the moribund patient at 3 o'clock in the morning with minimal information. Then you discover later they've got lung cancer. You know it's a waste of time. … I find quite frustrating because we spend a lot of time talking, you've got to talk to the family, you've got to talk to the patient*". Intensivist, Decision 2, Hospital 3<br><br>The referral might be manipulated: *"Interviewer: They called and said that the patient was tiring and you said, 'They always say that they are tiring'. ICU-consultant: Yes A&E use certain words and certain phrases to put the anti-up so we've got to go and do something."* Intensivist, Decision 6, Hospital 1

Table 4. Sources regarding clashing values justifying the cost of delaying decision. Values= CAPITALS, arguments and norms attached to values=underlined, sources i.e. quotations(*in italics)* underpinning views and field-notes as responses to the question: Does the benefit to the patient of getting the decision right justify the cost to the patient of a delay in making the decision?

**A. As much information as possible should be gathered before making a decision that is in the patient's best interests**	**B. The decision should be expedited to provide immediate relief of symptoms and reduce potential harm from treatment delay**
PREVENT LONG-TERM SUFFERING OF NON-BENEFICIENT ICU-ADMISSION<br>Symptom relief should be avoided to prevent risk for non-beneficial life-support: "*She was really struggling to breath, she wasn't tolerating oxygen very well, she was really struggling, she was just about maintaining her airway but she was very agitated, she was thrashing around a lot, but we weren't keen to intubate her."* Intensivist, Decision 4, Hospital 3<br><br>If clinician skilled in responsive interaction, it is of long-term beneficence to ask patients sensitive questions: "*He [the patient] understood what the doctor said. Oh, well he was the best doctors I've seen, he was brilliant, he was clear, he listened to dad. I just found him helpful and listening and I think he was good for my dad*" Daughter, Decision 6, Hospital 3<br><br>*"What I realised is that most of the reluctance in asking a question is not on the patient's side, it's usually on the physician's side. So what you feel is an odd question to ask you will think that the patient will feel uncomfortable answering. "* Intensivist, Decision 3, Hospital 1<br><br>PROTECTION OF PROFESSIONAL IDENTITY<br>Painful procedures are necessary for informing the decision: The intensivist stands silent by the bed looking at the junior doctor trying to insert an arterial line on the patient's wrist [the medical registrar looks distressed]. The patient breathes heavily, falls asleep and wakes up repeatedly and then looks constantly at her left wrist, now with several needle pinpricks and bruises. The intensivist turns to me: *'I really need a gas'* Field notes, Decision 2, Hospital 2<br><br>Fear being judged by peers if wrong decision: " *… if you admit them to ICU and they fail to benefit, you can feel as if you're being judged by your peers as, as to have made a wrong admitting decision*." Intensivist, Decision 4, Hospital 2<br><br>PERSON-CENTREDNESS ASKING PATIENTS VITAL QUESTIONS<br>Ethical and legal duty to bring up ceiling of care with the patient at first encounter: "*I have made it a practice when the last NICE guidance came out that ceiling of care decisions should be made at the time of first review. So what I started to do was that irrespective of how well or unwell you were I would ask you what you wanted to do in case you needed to be resuscitated"* Intensivist, Decision 3, Hospital 1<br><br>Presuppose that the patient may have capacity, therefore should all patients be asked about their wishes: "*The patient was still fairly lucid and it's not always the case. … I felt that she had capacity at that point in time and therefore she was able to make a decision*." Intensivist, Decision 2, Hospital 3	PREVENT SHORT-TERM SUFFERING BY ADMISSION<br>Stressful questions and procedures should be postponed to ICU, waiting for return of capacity: *"We did the right thing but in some ways it feels a bit strange to admit a man who you know, the bottom line is he is going to die from cancer and probably fairly soon. … He was able to talk with the next day. … we were quick glad of the opportunity to discuss with him and he went, "No I don't want to bleed to death, thank you very much"* Intensivist, Decision 4, Hospital 5<br><br>SENSE OF SECURITY<br>The patient wants to lay the difficult decision in the doctor's hand: *"The doctors know what they're doing. It's no good saying to me, 'What do you think about it?' because I haven't got a clue. I could have said, "Well I don't know, and that could have been the difference between life and death so I don't think you should have to ask*." Patient, Decision5, Hospital 6<br><br>The severely ill lacks capacity for complex decision making: The patient is grey in face, have difficulty of talking, out of breath, panic in eyes, removes the oxygen mask off repeatedly, tries to sit right up in agony. … The junior Intensivist asks the patient rapid questions about functional status, which he says yes to all of them [giving impression that he means he is in good health, denied by family later]. Field notes, Decision 5, Hospital 5<br><br>Inexperienced professionals should be hindered from inducing distress:<br>*"[Researcher:] You were occupied with the arterial line insertion. Did that affect the communication? [Medical registrar:] Yes certainly, so I mean that was sort of an unfortunately tricky procedure so may be where a little bit of the ego of the physician would come into play."* Junior referring doctor, Decision 2, Hospital 2<br><br>Relief of distress may give patient emotional and physical strength to survive: A nurse brings in the granddaughter, whom has long time been sitting outside the resuscitation room. She approaches the patient, takes her hand and strokes her over the head. The patient smiles [The patient seems happy to see her, seems to calm down, after a while resting her head on the stretcher, nodding off, respiratory rate lowers and the pulse on the screen]. Field notes, Decision 1, Hospital 4

Table 5. Sources of clashing values regarding lack of clarity of valuable input for the decision for the incapacitated patient together with arguments and quotations/field-notes as responses to the question: To what extent should the intensivist gain others' input or views before making a decision?

**A. The intensivist should make a clinical decision based on their own assessment without the need to seek the views of others.**	**B. The intensivist should invest time to gain multiple input in order to make a decision.**
PROFESSIONAL INDEPENDENCE<br>An experienced intensivist has discernment to represent the patient: " *I didn't want him[referring doctor] to make the decision on my behalf, not coming with the answer before I'd had time to vocalise my thoughts on the matter … that doesn't cloud my own judgement. … I made this decision independently. I didn't feel that I needed peer support for that*." Intensivist, Decision 4, Hospital 2<br><br>Other doctors lack knowledge what ICU cannot achieve: "*Most physicians lack, as opposed to the intensivist, experience and an understanding of what ICU- care can do for you. It's not a magical land where everything will be fixed miraculously; there are limits to what can be done*." Ref. med. registrar, Decision2, Hospital 2<br>*"It's easy for the teams to say, 'We'll get intensive care to come and they can take you to ICU,'. Actually it may be absolutely the wrong thing to do because putting someone on a ventilator is actually in some places a death sentence or they will never come off that ventilator."* Intensivist, Decision1, Hospital 4<br><br>Referring doctor may not be familiar with the patient: "*I was talking to one person and then it turns out that he's from A&E and actually the ward doctor is another person. … it was not really clear who knows the patient best and who is actually responsible for this patient."* Intensivist, Decision 7, Hospital 3<br>"*The referring teams, bless their cotton socks, don't have an adequate grasp of the medical facts and history of their patients. … they're very busy, the patient's only just arrived, there's no one else around to tell them, or it's completely outside of their clinical practice*.*"* Intensivist, Decision 4, Hospital 5<br><br>Family have unrealistic wishes what ICU can achieve: "*The problem is that the families have unrealistic expectations of the outcomes. That's partly driven by things on the media."* Intensivist, Decision 2, Hospital 1<br><br>TIME TO JUDGE<br>Decisions might be delayed if involving others: *"[Researcher:] Did you communicate with the doctor on the ward? [Intensivist:] No and under these circumstances I wouldn't necessarily do so as a time constraint."* Intensivist, Decision 3, Hospital 2<br>More time than others to make considerations*: "I have the luxury of time when I go and see a patient. My average time I spend with a patient is 40 minutes, when considering a non-admission.*" Intensivist, Decision 3, Hospital 5<br><br>SENSE OF SECURITY FOR FAMILY NOT BEING INVOLVED<br>Families trust the intensivist to make the decision: *"[Res:] Were you involved in any decisions? No, no, that was fine because I'm not a medical person so! That doctor seemed very knowledgeable about the whole."* Son, D3, H 4<br><br>Family might be in an avoidance- and emotionally unstable state not representing the patient's wishes: "*I have said at times if you know I was really ill, a burden, haven't got anything coming for me, I would rather they treated somebody else but my son says don't talk so silly! He doesn't like talking about things like that*'" Patient, Decision 4, Hospital 3 "That's *difficult because you are in a bit of turmoil, you don't take in everything they're sayin and it would be better if they were able to say to you, 'Right this happened, we are now moving her'. I couldn't make the decision as whether to move to intensive care or… hu!"* Son, Decision 4, Hospital 3	HOLISTIC APPROACH<br>To ensure ethical decision making, different perspectives are needed:<br>*"Age, maturity, being street wise, I don't know what it is that makes you more accepting of your own limitations and your own inabilities to make the right judgement, dealing with uncertainty for me is one of those qualities of a great physician, helping families and patients to deal with uncertainty. … I don't think that you're taught that, I think you learn that over time and that's why I expect my views now, more inclusive and different."* Intensivist, Decision 4, Hospital 4<br><br>Referring doctor need to share perspectives with intensivist<br>*"… a second pair of eyes and ears is very helpful and all the interaction with the team today. She was seen by the appropriate team, the extra info that's adjusted her case has been gleamed*" Referring doctor, Decision 3, Hospital 1<br><br>CONSISTENCY<br>Risk of arbitrary decisions if other perspectives are not sought, due to intensivist's personal biases of being hawks or doves :<br>Dove: *"I think I've become a bit more cautious and a lot more optimistic about what we can provide on intensive care for patients. I'm probably very eager to admit and I'd probably admit patients that my colleagues wouldn't … I think I'm on the softer side*" Intensivist, Decision 4, Hosp. 1<br>Hawk *"At the beginning I felt more reluctant to say no which is slowly with that confidence you also see what happens with the patient in your care and then you make a decision. If you have to say no you say no."* Intensivist, D 7, Hosp. 3<br><br>PERSON-CENTREDNESS BY INVOLVING FAMILY<br>Family can contribute despite distress: "*I mean I understand it is a question [regarding DNR] that you have to ask, because people have different views. I understand that it has to be asked but it's quite distressing. [Researcher:] Was it good that he asked you about it or shouldn't he have? [Daughter:] No I think he should ask because, if dad had strong views and I knew about it then I do think it is something that should be asked.*" Daughter, Decision 3, Hospital 1<br><br>Patient have a voice through family: *"And then we went in to speak to the wife and she said that he has said to his family that he would never want to be resuscitated if ever he needed it and that he would never want to be in a persistent vegetative state*." Intensivist, Decision 4, Hospital 6

Table 6. Clashing values regarding duty to patients as responses to the question: Should the intensivist have an ongoing duty of care to patients reviewed but not admitted to ICU?

**A. The patient needs continued ICU-expertise on ward**	**B. The intensivist's duty of care for the patient ends with the decision**
SHORT-TERM PATIENT SAFETY<br>Follows up treatment suggestions, due to difficulty to anticipate illness trajectory: Intensivist communicating with patient: "I will review in a couple of hours again" "we will make sure you are safe, we have a plan now, maybe we will give you more oxygen". Field-notes Intensivist, Decision 1, Hospital 5. Interview: *"When I first met him I laid down very clear criteria for his oxygenation is below a particular limit, but today when I went and did a blood gas on him, his oxygenation was very poor. Clinically he was not where I wanted him to be 48 hours down the line."*<br><br>Compensates for incompetence or lack of staff on the wards: "*So that would be a greater staff to patient ratio, more frequent observations, one of the issues with him was that his catheter was inserted in the morning and it was only first checked at 3pm*"<br>Referring medical registrar, Decision 6, Hospital 3<br><br>"*He* [the intensivist] *reviewed her at 11 o'clock* [pm] *before he went home and asked his team to keep an eye on her overnight, so that she had a much higher level of medical oversight. The important thing is about having enough people around to make clinical assessments of patients so that if they are deteriorating they're picked up in time".*<br>Nephrology consultant, Decision 7, Hospital 6<br><br>Assumes responsibility for severely ill patients wherever they are: *"So I would like to think that we don't restrict intensive care even if we don't have room in our intensive care unit. … I would arrange for that patient to receive appropriate care wherever that be. So for that reason the decision to admit to ICU to me becomes an artificial construct. I'd like to think that we should deliver the care that is appropriate, the judgement not whether they should come to ICU."*<br>Intensivist, Decision 4, Hospital 4<br><br>SHORTTERM PROFESSIONAL SECURITY OF THE REFERRING TEAM<br>Gives support to uncertain junior doctors: "She [the intensivist] *was mirroring my own thoughts, not just what's happening here and now. She clearly had a very pragmatic view of the future and so she seemed to be weighing up and then communicating to me of what her concerns were*."<br>Referring medical registrar, Decision 2, Hospital 2<br><br>Gives end-of-life support management to referring team<br>"*I thought that trying to meaningfully treat him and get him home again was unlikely and so I said no to admission. I asked my junior to escalate that to the medical team and then to make sure that we were adequately analgised and kept comfortable and that DNR should be completed.* Intensivist, Decision4, Hospital 5	LONG-TERM PATIENT SAFETY<br>Ward staff should be expected to have basic skills to care for severely ill patients: The senior intensivist is complaining about ED, that the junior intensivist has done too much job there. She says this is "*classic difficulties*" that ED and the wards can very well manage this kind of patient. "*She needs intravenous fluids, intravenous antibiotics, some oxygen and these are all things that should be able to be managed on the ward.... The trouble is the bigger we've got as an ITU and the more we have things like Outreach nurses, the wards get deskilled*" Field-notes, Decision1, Hospital 4<br><br>Wards should be staffed for caring for severely ill patients: "Cover over night is not great. It is 1 or 2 trained nurses, 1 or 2 agency nurses possibly, the ward has high agency. There is no onsite kidney specialist overnight and the ward is run mainly by general medical doctors, covering several floors. Nephrology consultant, Decision 7, Hospital 6<br><br>FAIRNESS REGARDING ACCESS TO ICU-EXPERTISE<br>Non-medical reasons of intensivist input may be unfair to other patients: *"…you might argue that if they are under-staffed then it's not safe for him on the ward but equally … if an emergency comes from outside how do we justify who gets the help.*" Intensivist, Decision 6, Hospital 5<br><br>LONG-TERM SECURITY OF REFERRING TEAM<br>Important that ward staff are confident: The staff nurse interrupts the intensivist and junior referring doctor. She reports about *"awful urine"* and starts to ask the intensivist questions. Then she tells the junior doctor that he has to do this and that. The nurse seems to take responsibility and guides the doctor what to do and makes sure to get information from the intensivist how to manage the patient. Field-notes, Decision 6, Hospital 3<br><br>Referring team knows the patient best for confident end-of-life communication: The intensivist approaches the night nurse: "*Do you know what language she speaks?*" Nurse: "*Mandarine*". [Intensivist]" I will bleep the surgical registrar". He answers the telephone: " *Will you pass a message discussed with my consultant … She is for ward based care only. I don't think she will survive, so interpreter is important, to talk to family*". Field-notes, Decision 8, Hospital 2
